# Beyond Biomaterials: Engineering Bioactive Hydrogels as Immuno-Mechanobiological Niches for Osteochondral Regeneration

**DOI:** 10.3390/gels11080658

**Published:** 2025-08-19

**Authors:** Francesca Semeraro, Valentina Rafaela Herrera Millar, Lucia Aidos, Mirko Sergio, Lorenzo Impieri, Giuseppe Michele Peretti, Laura Mangiavini, Alessia Di Giancamillo, Nicolò Rossi

**Affiliations:** 1 Residency Program in Orthopedics and Traumatology, University of Milan, 20141 Milan, Italy; francesca.semeraro@unimi.it (F.S.); lorenzo.impieri@unimi.it (L.I.); 2 Department of Biomedical Sciences for Health, University of Milan, 20141 Milan, Italy; valentina.herrera@unimi.it (V.R.H.M.); giuseppe.peretti@unimi.it (G.M.P.); laura.mangiavini@unimi.it (L.M.); 3 Department of Veterinary Medicine and Animal Sciences, University of Milan, 26900 Lodi, Italy; lucia.aidos@unimi.it (L.A.); mirko.sergio@unimi.it (M.S.); 4 IRCCS Ospedale Galeazzi Sant’Ambrogio, 20161 Milan, Italy

**Keywords:** osteochondral regeneration, osteoimmunomodulation, mechanobiology, osteochondral unit, tissue regeneration, hydrogel, bioactive hydrogels

## Abstract

Osteochondral regeneration remains a major clinical challenge due to the complex architecture and biomechanical demands of the osteochondral unit. Bioactive hydrogels have emerged as promising materials capable of supporting repair through their capacity to mimic the extracellular matrix (ECM), enable cell encapsulation, and deliver bioactive cues. However, recent insights reveal that simply engineering hydrogels for structural and cellular support is insufficient. A new paradigm is emerging—one that embraces the complexity of the osteochondral niche by integrating immunomodulatory and mechanobiological cues into biomaterial design. In particular, the hydrogel’s capacity to modulate macrophage polarization and support the immunoregulatory function of mesenchymal stem cells (MSCs) is critical to orchestrate regenerative outcomes. Simultaneously, the mechanical properties of hydrogels—such as stiffness, porosity, and viscoelasticity—can profoundly influence stem cell fate and local tissue morphogenesis. This review discusses recent advances in hydrogel-based strategies for osteochondral repair, highlighting the interplay between immunological signals and the mechanical microenvironment, and calls for a shift from reductionist tissue-engineering approaches to systems-level design of tunable, immuno-mechanobiological microenvironments.

## 1. Introduction: From Scaffold to Niche

Damage to osteochondral tissue, involving both articular cartilage and subchondral bone, is challenging to repair. While bone tissue possesses a significant regenerative capacity, articular cartilage has limited potential for self-repair [1]. While various surgical interventions are available, such as microfracture or osteochondral autografting, long-term results are often suboptimal, particularly in restoring the zonal architecture and mechanical integrity of native tissue [2].

Hydrogels are three-dimensional (3D) hydrophilic polymers capable of retaining water and biological fluid due to the presence of hydrophilic groups such as -COOH, -OH, -CONH_2_, -CONH, -SO_3_H, and -NH_2_ [3,4]. Hydrogels have been at the forefront of biomaterials research in this context. Their high water content, injectability, and structural similarity to the extracellular matrix have made them attractive platforms for cell encapsulation, biomolecule delivery, and guided tissue growth [5,6]. Hydrogels may be designed to have immunomodulatory properties that allow them to change the microenvironment, as in the case of diabetic foot ulcers [7], or even present antimicrobial properties that promote the healing of bacteria-infected wounds [8], including in cases of multidrug-resistant bacteria [9]. Many natural and synthetic hydrogel formulations have been tailored for improved biocompatibility and functionality in osteochondral repair applications, with several demonstrating promising preclinical outcomes [10].

Despite these advances, the field is increasingly recognizing that structural mimicry alone is not enough. Traditional strategies in tissue engineering have often taken a reductionist view—reconstructing isolated tissue components without fully considering the complex, dynamic environment in which regeneration occurs. A more effective approach involves treating the biomaterial not just as a structural support but as a regenerative microenvironment, capable of interacting with multiple cellular and biochemical systems [11].

One key component of this environment is the immune system. Tissue injury inevitably elicits an inflammatory response, and the success of repair is closely tied to how the immune system—particularly macrophages—responds to biomaterials. Hydrogels can be designed to shift macrophage behavior from pro-inflammatory (M1) to pro-regenerative (M2) phenotypes, a process known as osteoimmunomodulation [12,13,14]. Furthermore, mesenchymal stem cells (MSCs), often delivered via hydrogels, exert potent immunomodulatory effects through their secretome, adding another layer of complexity to this interaction [15,16].

Simultaneously, mechanical cues have been shown to influence cell behavior and differentiation, particularly in stem cell-based therapies [17,18,19]. The mechanical properties of hydrogels, such as stiffness, elasticity, and matrix architecture, can regulate mechanotransduction pathways that guide chondrogenic and osteogenic differentiation. Thus, by tuning these physical properties, hydrogel design can exert profound effects on the cellular responses that underpin regeneration.

This review aims to synthesize these two emerging domains—immune modulation and mechanobiological tuning—within the context of hydrogel design for osteochondral regeneration. We advocate for a holistic approach that acknowledges and engages with the biological complexity of the osteochondral niche. Through this perspective, we explore how smart biomaterials, defined as materials able to modify their functional or physical properties when exposed to environmental changes or external stimuli, can be harnessed not just to support regeneration but to actively orchestrate it.

In this review, the term ‘hydrogel-based scaffold’ will be used exclusively to refer to three-dimensional hydrogel systems designed to support cell growth and tissue formation. When referring to composite systems where hydrogels are used as coatings or functional layers over other core materials (e.g., bone grafts or membranes), this will be explicitly stated to avoid ambiguity. The term ‘scaffold’ alone will be avoided to ensure terminological clarity.

## 2. The Evolving Landscape of Hydrogels for Osteochondral Regeneration

Hydrogels have emerged as versatile materials in the context of osteochondral repair due to their high water content, structural similarity to the native extracellular matrix (ECM), and the capacity for controlled delivery of cells and bioactive molecules. Broadly, hydrogels can be categorized into natural, synthetic, and hybrid types, each with distinct properties that influence their biological performance (Table 1).

### 2.1. Natural Hydrogels

Natural polymers such as collagen, gelatin, hyaluronic acid (HA), chitosan, and alginate offer inherent biocompatibility and bioactivity. These naturally present biochemical motifs that interact with cellular receptors such as integrins, thereby promoting cell adhesion, proliferation, and lineage-specific differentiation [20,21]. HA-based hydrogels, for example, closely resemble the composition of native cartilage ECM and have been shown to support chondrocyte viability, glycosaminoglycan synthesis, and type II collagen expression.

Collagen–HA composites have been investigated in subchondral defect models and shown to improve osteogenesis by upregulating osteogenic markers and stimulating new bone formation [22]. Gelatin, a denatured form of collagen, offers additional advantages due to the presence of cell-interactive sequences such as RGD peptides. When functionalized (e.g., gelatin methacryloyl, GelMA), it enables photo-crosslinking and fine-tuned mechanical stability, making it particularly suitable for 3D bioprinting. GelMA–HA composite hydrogels have facilitated osteoblast attachment and mineralization, providing a robust platform for osteochondral material fabrication [23,24]. Functional hydrogels present, however, a considerable cost, which may represent an economic barrier for clinical adoption.

Chitosan, recognized for its hemostatic and antimicrobial properties, has been combined with HA, collagen, or nano-hydroxyapatite (nHA) to form composite hydrogels that enhance MSC osteogenic differentiation and mineral deposition [25]. In preclinical models, chitosan–HA systems implanted in articular defects have demonstrated promising outcomes, including enhanced type II collagen expression and hyaline-like cartilage regeneration. Meanwhile, alginate, despite its ability to maintain chondrocyte phenotype and support matrix synthesis, is limited by poor mechanical strength and a lack of cell adhesion sites. To address these limitations, alginate is frequently blended with more mechanically robust or bioactive materials, such as HA, gelatin, or ceramic fillers.

Nonetheless, several limitations restrict the standalone application of natural hydrogels in osteochondral repair. Variability in biological source and processing leads to inconsistency between batches, and degradation rates are often unpredictable [26]. Furthermore, their mechanical performance under physiologic compressive loading is typically insufficient—particularly for collagen, gelatin, HA, and alginate-based systems [5]. While composite strategies (e.g., collagen–HA or chitosan–nHA hybrids) can partially overcome these deficiencies by improving mechanical strength and biological signaling, they also introduce greater complexity in fabrication and regulatory approval. Therefore, to achieve clinical translation, natural hydrogels are increasingly being integrated into multilayered or hybrid constructs that combine mechanical reinforcement with biofunctionality tailored to each osteochondral zone.

### 2.2. Synthetic Hydrogels

Synthetic polymers such as poly(ethylene glycol) (PEG), poly(vinyl alcohol) (PVA), polylactic acid (PLA), and poly(lactic-co-glycolic acid) (PLGA) hydrogels offer precise modulation of hydrogel mechanics, crosslinking density, and degradation kinetics, providing superior design control compared to natural polymers [27]. The mechanical performance of purely synthetic hydrogels such as PVA, while promising in mimicking cartilage properties, may fall short in supporting long-term osteochondral integration without reinforcement.

PEG-based hydrogels, while inherently bioinert, can be functionalized with cell-adhesive peptides such as RGD or degradable motifs to enable cell attachment and protease-mediated remodeling—features essential for cartilage and bone [28]. PLA-hydrogels are emerging as interesting materials; specifically, they offer a high surface area compared to volume, a high degree of porosity, and strong mechanical properties. Moreover, they can be supplemented with growth factors or antimicrobial substances [29]. PLA-hydrogels are thermo-responsive materials that remain soluble at room temperature but form gels at body temperature (37 °C).

For example, PLGA–hydrogel composites reinforced with nano-hydroxyapatite supported mesenchymal stem cell (MSC) viability, inducing smooth and hyaline-like cartilage formation with abundant glycosaminoglycan and collagen II deposition in a rat osteochondral defect model [30]. Similarly, a hybrid PLGA-gelatin-chondroitin-HA material increased MSC proliferation and extracellular matrix synthesis, and improved cartilage repair in rabbit models when compared to the PLGA-only platform [31]. Despite their tunability and structural robustness, synthetic hydrogels lack inherent bioactivity, necessitating the incorporation of natural elements or bioactive fillers. For instance, composite PLGA/gelatin supports demonstrated improved chondrogenic matrix production over PLGA alone in vitro and in vivo. However, synthetic polymers often degrade into acidic byproducts (e.g., lactic and glycolic acids), which can impair local pH and hinder tissue regeneration if not buffered appropriately. Mitigation strategies have been applied, such as the addition of nano-hydroxyapatite (nHA), which resulted in appropriate degradability in vitro [32]. The synergistic osteogenic and anti-inflammatory effect of aspirin and (nHA) was also verified in mouse bone tissue [33]. 

### 2.3. Hybrid and Composite Hydrogels

In this section, hybrid and composite hydrogels are examined. These hybrid systems offer significant advantages in tissue regeneration, due to their combination of mechanical properties of scaffolds with the biochemical characteristics of hydrogels.

To harness the synergistic benefits of both natural and synthetic polymers, hybrid hydrogels have emerged as robust platforms for osteochondral regeneration. These systems unite the cell-adhesive and enzymatically degradable features of natural biopolymers with the precise tunability and mechanical integrity of synthetic networks. A prominent example is gelatin methacryloyl (GelMA), which retains RGD-binding sites from gelatin and enables photopolymerization to spatially tune mechanical properties. GelMA’s versatility is exemplified by its incorporation in 3D-bioprinted biphasic constructs, such as PCL/GelMA constructs seeded with mesenchymal stem cells and chondrocytes [34].These materials achieved cartilage repair with tissue mechanics comparable to native cartilage in rat osteochondral defect models. The enhancement of GelMA’s osteogenic capacity has been demonstrated through integration of mineralized nanofillers. For instance, a gradient triphasic GelMA–nano-hydroxyapatite (nHA) structure recreated the mechanical and compositional transition from cartilage to bone [24]. This construct exhibited continuous pore-size gradients, a Young’s modulus (~180 kPa) similar to native subchondral bone and supported simultaneous chondrogenesis and osteogenesis.

Hybrid systems combining synthetic polymers and inorganic components also show promise. For example, poly(lactic-co-glycolic acid) (PLGA) constructs embedded with gelatin, chondroitin sulfate, HA, and BMP-2 have been demonstrated to promote robust cartilage and subchondral bone regeneration in rabbit osteochondral lesions [35]. Likewise, biphasic hydrogel–bioactive glass composites have supported osteochondral defect repair in rabbit models, although these systems may require careful compartmentalization to prevent antagonistic interactions between bone and cartilage phases [36].

A recent advance includes GelMA material reinforced with mineralized hydroxyapatite nanofibers. These composites improved the compressive modulus, controlled degradation, and delivered enhanced bone regeneration in critical-sized defects, though optimal filler concentrations must be identified to avoid compromising gel stability [37].

Limitations of these hybrid platforms include the potential for phase separation, variable filler dispersion, and challenges in achieving consistent crosslinking interfaces between layers. As complexity increases—from biphasic to gradient and even triphasic constructs—manufacturing reproducibility and mechanical reliability become critical factors, particularly for clinical translation.

### 2.4. Functionalization for Bioactivity

Beyond mechanical and structural optimization, hydrogels can be functionalized with biological signals such as growth factors (e.g., TGF-β, BMPs, VEGF), small molecules (e.g., kartogenin), or peptides (e.g., IKVAV, YIGSR) to direct specific cell behaviors. Strategies for spatiotemporal control of these cues—such as sequential or localized release—have shown promise in guiding the progression of regeneration across cartilage and bone interfaces [38].

Among biological signals, growth factors represent one of the most studied classes of biomolecular regulators. In particular, members of the TGF-β superfamily—notably TGF-β1 and TGF-β3—have demonstrated potent chondrogenic effects. These cytokines stimulate mesenchymal stromal cells (MSCs) and other progenitors to differentiate into chondrocytes and synthesize key cartilage ECM components such as type II collagen and aggrecan [39]. Hydrogels incorporating TGF-β1/3—whether via covalent tethering, affinity-based loading, or encapsulated microparticles—have shown enhanced cartilage formation in vitro and in vivo [40]. Strategies such as localized gradients or layer-specific loading are particularly attractive for osteochondral applications, allowing preferential stimulation of chondrogenesis in the superficial zone and osteogenesis in the deeper region [41]. Subchondral bone repair requires coordinated angiogenesis to restore vascularized bone tissue and support osteochondral integration. Although pore interconnectivity is often emphasized in material design, it must be functionally linked to measurable vascular ingrowth metrics [42]. One promising approach is the controlled delivery of vascular endothelial growth factor (VEGF), a potent angiogenic molecule. Studies in large animal models such as pigs have demonstrated that local VEGF administration significantly enhances neovascularization and bone repair at the osteochondral interface, highlighting the translational relevance of such strategies [43]. A promising approach involved the use of a 3D-printed porous titanium structure combined with a thermosensitive collagen hydrogel loaded with VEGF, demonstrating prolonged angiogenic factor release, enhanced local neovascularization, and improved osseointegration in preclinical models [42]. Another study focused on the meniscus: endostatin (COL18A1) incorporated into 3D fibrin hydrogels promoted the fibro-chondrogenic differentiation of neonatal porcine meniscal cells and modulated angiogenesis by influencing VEGF expression, improving cell density, glycosaminoglycan production, and the expression of markers such as COL1, COL2, and SOX9 after 21 days in culture [44]. These findings emphasize the need to integrate VEGF release kinetics with matrix architecture to maximize vascular infiltration and regenerative outcomes in osteochondral applications.

More recently, bioactive hydrogels have been engineered to respond dynamically to environmental stimuli, such as pH, enzyme activity, or reactive oxygen species, enabling on-demand release of therapeutic agents in response to tissue needs [45].

## 3. Immunomodulatory Hydrogels: Engineering the Healing Response

**Figure 1:**
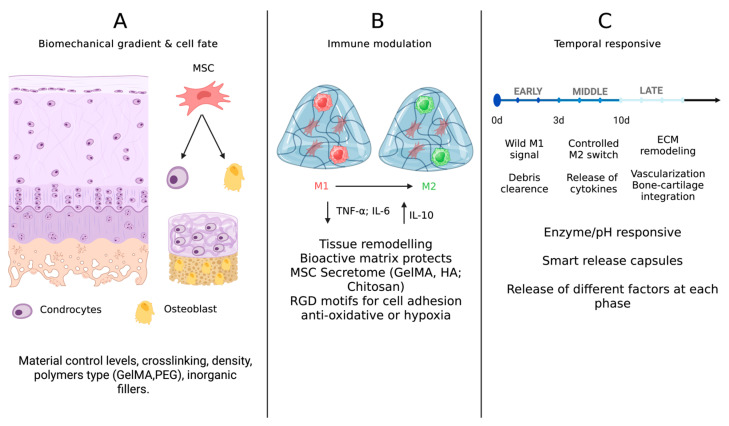
(**A**) Biochemical gradient and cell fate; (**B**) immune modulation; (**C**) temporal responsiveness. Created in BioRender. Sergio, M. https://BioRender.com/ts51w3y (accessed on 9 August 2025).

Traditional hydrogel-based tissue engineering has largely focused on creating biomimetic environments conducive to cell survival, proliferation, and differentiation. However, the initial immune response to any biomaterial implantation is now recognized as a critical determinant of tissue regeneration outcomes. In the osteochondral context, where both cartilage and subchondral bone interact with resident immune cells, engineering hydrogels to modulate immune activity—a process termed osteoimmunomodulation—has emerged as a key strategy [12,46]. The issues of the following sub-chapters are summarized in Figure 1.

### 3.1. Macrophage Polarization: From Inflammation to Regeneration

Macrophages, central players in innate immunity, exhibit remarkable plasticity and can shift between pro-inflammatory (M1) and anti-inflammatory, pro-regenerative (M2) phenotypes depending on the environmental cues [47]. This polarization spectrum is particularly relevant in osteochondral healing, where prolonged M1 activation leads to chronic inflammation and tissue degeneration, while timely M2 transition supports repair, angiogenesis, and remodeling [48] Hydrogels can be engineered to actively modulate macrophage behavior through surface chemistry, degradation products, or controlled release of cytokines (e.g., IL-4, IL-10, TGF-β1). For instance, regarding wound healing, hydrogels incorporating ECM-mimetic motifs or oxidation-sensitive linkers can attenuate inflammatory responses and promote macrophage-mediated tissue repair [49].

### 3.2. MSC Secretome and Indirect Immunomodulation

Mesenchymal stem cells (MSCs) embedded within hydrogels for osteochondral repair act not only as progenitor cells but also as critical regulators of the immune microenvironment, particularly through their influence on macrophage polarization. MSCs secrete a repertoire of anti-inflammatory molecules—including prostaglandin E_2_ (PGE_2_), TNF-stimulated gene-6 (TSG-6), indoleamine 2,3-dioxygenase (IDO), and interleukin-1 receptor antagonist (IL-1Ra)—that orchestrate the shift of macrophages from the pro-inflammatory M1 phenotype toward the reparative M2 state [50]. This phenotypic switch is essential for resolving inflammation and promoting tissue regeneration in osteochondral lesions.

MSC-derived PGE_2_ activates the EP2 and EP4 receptors on macrophages, triggering the cyclic AMP (cAMP)/protein kinase A (PKA) signaling pathway, which suppresses pro-inflammatory cytokine production (e.g., TNF-α, IL-6) and induces expression of M2 markers such as CD206 and arginase-1. Similarly, TSG-6 modulates macrophage activity by inhibiting NF-κB signaling, thereby reducing the secretion of matrix-degrading enzymes and pro-inflammatory mediators, which preserves cartilage matrix integrity [51]. IDO exerts immunosuppressive effects by depleting tryptophan in the microenvironment and producing kynurenine metabolites, which promote regulatory T cell expansion and further reinforce macrophage M2 polarization through the aryl hydrocarbon receptor (AhR) pathway [50]. IL-1Ra competitively inhibits IL-1β signaling, blocking MAPK and NF-κB pathways in macrophages, thereby preventing the amplification of inflammation and supporting tissue repair processes [50].

Hydrogel encapsulation strategies that preserve MSC viability and mechanotransduction—such as modulation of material stiffness and oxygen tension—can enhance this immunomodulatory secretome. For instance, hypoxic preconditioning of MSCs upregulates hypoxia-inducible factor 1-alpha (HIF-1α), which increases secretion of vascular endothelial growth factor (VEGF) and transforming growth factor-beta (TGF-β), further promoting M2 macrophage polarization and chondrogenic differentiation. Additionally, integrin-mediated focal adhesion kinase (FAK) signaling activated by hydrogel microenvironments enhances MSC paracrine activity, fostering a pro-regenerative macrophage phenotype [52].

Despite these advances, variability in the composition of MSC secretome, the limited in vivo persistence of MSCs, and the complexity of the joint microenvironment remain significant challenges. Achieving precise control over macrophage polarization dynamics through hydrogel design and MSC modulation is critical for translating these immunoregenerative strategies into effective osteochondral therapies.

### 3.3. Temporal Immunomodulation and “Immuno-Instructive” Materials

Emerging strategies involve temporal control over immune modulation. The success of these approaches relies on the timing of immune modulation: a delayed or incomplete switch from M1 to M2 macrophages has been associated with impaired extracellular matrix deposition, vascularization failure, and prolonged inflammation [53]. To emulate this dynamic, hydrogels are being engineered with sophisticated release profiles and degradation-sensitive architectures that provide sequential delivery of bioactive molecules or expose specific biofunctional groups in response to environmental cues. These “immuno-instructive” hydrogels represent a paradigm shift from passive structural supports to dynamic, biointeractive systems capable of guiding cellular behavior and immune cell crosstalk in a spatiotemporally controlled manner [54]. Some strategies also incorporate “immune training” elements by pre-conditioning materials with cytokines or co-culturing with MSCs to imprint an immunomodulatory profile before implantation [55]. Others explore integrating mechanical cues or redox-sensitive motifs that act synergistically with cytokine release to further fine-tune macrophage behavior.

Challenges persist in precisely controlling the timing and dosage of factor release to accommodate patient-specific variability, as well as in managing the complex feedback interactions between immune cells and local tissue environments, which can lead to unpredictable outcomes. Moreover, scaling up the production of these advanced hydrogels while maintaining consistent quality, alongside navigating regulatory approval processes, remains a significant hurdle. Comprehensive in vivo studies and the ongoing optimization of these temporally controlled immunomodulatory systems are essential to fully realize their potential in effective osteochondral regeneration.

## 4. Mechanobiology in Hydrogel Design: Sculpting Cell Fate Through Force

**Figure 2:**
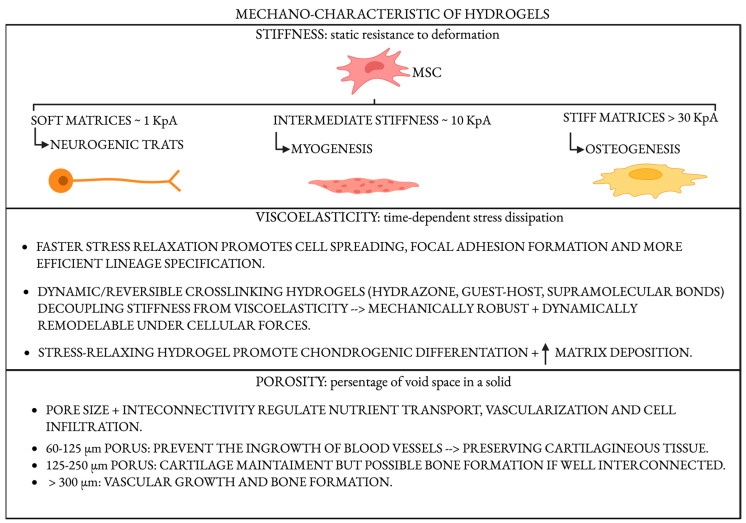
Illustrative scheme of mechano-characteristics of hydrogel. Created in BioRender. Sergio, M. https://BioRender.com/gr2wiej (accessed on 9 August 2025).

Mechanical signals within the osteochondral interface serve as pivotal regulators of cellular behavior and tissue morphogenesis. Cartilage and subchondral bone experience markedly different mechanical environments: cartilage endures complex compressive, tensile, and shear forces within a viscoelastic matrix, while subchondral bone is exposed primarily to high-magnitude compressive loads within a mineralized and rigid framework. The resident cells—chondrocytes, osteoblasts, and mesenchymal stem cells (MSCs)—are equipped with intricate mechanotransduction machinery that converts mechanical stimuli into intracellular biochemical signals. This process governs gene expression, lineage commitment, and extracellular matrix (ECM) remodeling, orchestrating tissue regeneration [56,57]. Central to mechanotransduction in the osteochondral niche are integrin-based focal adhesions, cytoskeletal tension, and mechanosensitive ion channels, notably Piezo1/2 and TRPV4 [58]. The Hippo pathway effectors YAP and TAZ serve as master mechanosensors that integrate matrix stiffness and mechanical stress into transcriptional programs. In the osteochondral interface, the steep gradient of mechanical properties results in spatially regulated YAP/TAZ activity: low stiffness in the cartilage zone reduces nuclear localization, promoting chondrogenesis, whereas the stiffer mineralized bone promotes nuclear YAP/TAZ translocation and osteogenesis [59]. This zonal mechanotransduction and the associated gradient in lineage specification differentiate the osteochondral niche from more mechanically uniform tissues such as muscle or skin. Mechanical cues also influence the secretion of autocrine and paracrine factors—including prostaglandins, nitric oxide, and TGF-β/BMP family cytokines—that regulate osteoimmunomodulation and matrix remodeling, further underscoring the complexity of osteochondral mechanobiology [60]. An illustrative scheme is represented in Figure 2.

### 4.1. Stiffness: A Master Regulator of Lineage Commitment

Matrix stiffness influences stem cell fate. Pioneering work by Engler et al. [61] showed that MSCs cultured on soft matrices (~1 kPa) adopt neurogenic traits, intermediate stiffness (~10 kPa) favors myogenesis, and stiff matrices (>30 kPa) promote osteogenesis. This concept has been effectively translated to osteochondral tissue engineering, where gradient stiffness hydrogels or bilayered systems have been developed to replicate the mechanical heterogeneity of native tissues. These materials promote chondrogenesis in the softer, superficial cartilage-mimicking zones and osteogenesis in the stiffer, bone-facing regions.

To achieve precise control over stiffness, hydrogels such as methacrylated gelatin (GelMA) and polyethylene glycol (PEG)-based composites have been employed, enabling tunability through variations in crosslinking density or chemical functionalization. This enables the spatially defined mechanical properties necessary to guide zonal differentiation and the formation of integrated osteochondral constructs [62].

### 4.2. Viscoelasticity and Stress Relaxation

While stiffness provides a static measure of a material’s resistance to deformation, viscoelasticity—how a material dissipates stress over time—has emerged as an equally important factor influencing cellular behavior. Materials that exhibit faster stress relaxation promote cell spreading, focal adhesion formation, and enhanced lineage specification [63]. Recent hydrogel formulations with dynamic, reversible crosslinking (e.g., hydrazone, guest–host, or supramolecular bonds) allow the decoupling of stiffness from viscoelasticity, opening new design possibilities [64]. This breakthrough allows the design of hydrogels that are mechanically robust yet capable of dynamic remodeling under cellular forces.

Studies have shown that stress-relaxing hydrogels enhance chondrogenic differentiation and matrix deposition in encapsulated MSCs, making this property particularly relevant for cartilage regeneration [65]. Beyond the intrinsic material properties, the spatial organization and the ability to transmit biomechanical loads also profoundly influence osteochondral regeneration, bridging material mechanics and biological function.

### 4.3. Porosity, Architecture, and Load Transmission

Pore size and interconnectivity affect nutrient diffusion, vascularization, and cellular infiltration. Macro-porous and interconnected networks facilitate bone ingrowth, while smaller, denser networks help maintain chondrogenic environments [66,67]. Specifically, pores measuring 60–125 μm allow cell migration and cartilage formation while avoiding endochondral ossification. Pores between 125 to 250 μm promote chondrogenesis, unless they are well interconnected, in which case they can also support bone formation. Pores larger than 300 μm facilitate the development of a vascular network and, consequently, bone formation [68].

Additive manufacturing and sacrificial templating allow the fabrication of graded or zonally organized architectures that replicate native tissue structure [69]. In the osteochondral context, porous architectures must support limited vascularization in cartilage while promoting robust blood vessel ingrowth in the subchondral bone region. Optimizing pore size and interconnectivity enables a balance between mechanical integrity and biological function, allowing vascular invasion where needed, without compromising the load-bearing properties of the structure. Moreover, hydrogels must transmit and distribute physiological loads appropriately. If they are too soft, they fail to stimulate osteogenesis; if they are too rigid, they risk stress shielding or cell damage. Load-bearing composite hydrogels (e.g., reinforced with nanofibers, particles, or printed lattices) are being developed to provide the mechanical competence required for osteochondral interfaces.

## 5. Integrative Strategies: Toward Smart and Zonal Hydrogels

The osteochondral unit is a functionally graded interface composed of distinct yet interdependent zones: the articular cartilage, the calcified cartilage layer, and the subchondral bone. A major challenge in osteochondral regeneration is the replication of this hierarchical architecture within a single biomaterial system. In recent years, the development of zonal and integrative hydrogel strategies has gained attention, aiming to mimic the stratified organization of native tissue and provide the right cues to each compartment. Typically, the cartilage-facing region incorporates soft, viscoelastic matrices conducive to chondrogenesis, enriched with factors such as TGF-β isoforms or SOX9-activating peptides, while the subchondral bone region favors stiffer matrices loaded with calcium phosphates or BMPs to support osteogenesis. The calcified cartilage zone may function as a transitional interface with tunable mineral content and stiffness.

Zonal hydrogels are engineered to recapitulate the layered structure of osteochondral tissue by spatially organizing different materials, mechanical properties, and biological signals. One of the earliest examples of zonal design was described in 2009, when researchers created a bilayered model by isolating chondrocytes from different cartilage zones and encapsulating them in agarose at varying concentrations [70]. This construct was designed to reproduce the distinct biochemical and mechanical profiles of superficial and middle/deep cartilage layers [70]. More recently, Scalzone et al. [71] developed a sophisticated biphasic construct to mimic both cartilage and subchondral bone tissues. The cartilage compartment was engineered using a hydrogel composed of methacrylated gellan gum, chondroitin sulfate, and dopamine, loaded with Y201-derived chondrocytes. The bone compartment combined polylactic acid (PLA) with gelatin and nanohydroxyapatite, seeded with the same cell line to replicate bone matrix composition and mineralization. This model not only reflects the spatial and mechanical gradients of the osteochondral unit but also highlights the integration of tailored hydrogel chemistry for functional zonation.

However, a critical challenge that remains insufficiently addressed is the integration between these distinct zones, particularly the risk of delamination at the interfaces caused by abrupt differences in mechanical and biochemical properties. This delamination can compromise the structural integrity and long-term functionality of the construct, posing a significant barrier to clinical translation [72,73]. To address this issue, recent studies have begun to provide mechanical testing data supporting continuous gradient strategies. For example, Barkow et al. [74] experimentally characterized osteochondral gradient structures, demonstrating that continuous gradients improve force distribution and reduce delamination risk through enhanced shear strength and tensile properties [74]. Similarly, Brown et al. [75] showed that improved interfacial integration increases mechanical cohesion and shear strength between cartilage and bone layers. These findings reinforce the potential of continuous gradient designs to overcome delamination challenges; however, further mechanical testing is necessary to fully validate these approaches, as the number of comprehensive studies on mechanical performance at the osteochondral interface remains limited, highlighting the need for more focused research in this area. To overcome this, strategies incorporating continuous gradients of stiffness, porosity, and biochemical signals across the osteochondral hydrogel-based systems are being explored, aiming to mimic the native gradual transitions more faithfully rather than creating sharp bilayer boundaries. Such gradient designs improve interfacial bonding and reduce mechanical mismatch, promoting a more seamless integration between cartilage and bone compartments [76]. Advanced fabrication technologies, including gradient bioprinting and sequential crosslinking methods, enable precise spatial control over hydrogel composition and properties. At the same time, photo-initiated sequential crosslinking can create layered networks with tunable interfacial gradients to enhance mechanical cohesion [77]. In parallel, the development of “smart” or stimuli-responsive hydrogels has added a new dimension to hydrogel-based system design [45]. These materials are capable of adapting to environmental changes by releasing bioactive factors or altering their structure in response to specific stimuli, such as pH shifts, enzymatic activity, redox gradients, or mechanical loading.

## 6. Outlook

The evolution of osteochondral regeneration strategies from reductionist, cell- or ECM-centric models toward dynamic, system-level approaches represents a promising but inherently more complex path. Designing hydrogels as bioinstructive environments—capable of guiding immune responses, modulating mechanical signaling, and supporting spatially distinct yet interconnected tissue compartments—requires not only deeper biological understanding, but also a profound shift in how biomaterials are conceptualized, tested, and translated. The incorporation of multiple functionalities, such as immunomodulation, mechanosensitivity, and zonal architecture, increases the design space exponentially but also introduces significant challenges in terms of material fabrication, reproducibility, and regulatory approval. Ensuring consistency across batches of complex hydrogels—especially those containing gradients, responsive elements, or live cells—poses significant hurdles for standardization and upscaling.

From a translational perspective, hydrogel biocompatibility is well-supported locally in most preclinical models, with inflammation typically tied to specific chemistries or byproducts [78]. Degradation products appear to clear safely—particularly when hydrophilic components are used—though systemic organ-specific data remains limited. Functional lifespans range from weeks to months, depending on degradation mechanisms and matrix design [79]. Key translational hurdles include synchronizing scaffold degradation with tissue healing, preventing fibrotic encapsulation, and ensuring interface integration. These challenges are emphasized in recent reviews of multifunctional osteochondral hydrogels [11]. Moreover, the dynamic and patient-specific nature of osteoimmunological responses introduces biological variability that is difficult to model preclinically. Regulatory frameworks are not yet fully equipped to assess the safety and efficacy of multifunctional, responsive biomaterials that interact with host tissues in non-linear ways. From a translational perspective, integrating these hydrogels into surgical workflows, ensuring their mechanical integrity under joint loading, and predicting their long-term performance remain unresolved challenges. Additionally, the preclinical models typically used in cartilage or bone research often fail to capture the full physiological complexity of the osteochondral interface or the chronic inflammatory environment seen in human disease. Addressing these obstacles will require interdisciplinary collaboration, innovative in vitro and in vivo models, and new regulatory paradigms that can accommodate the biological and functional sophistication of next-generation biomaterials. Despite these hurdles, the field stands at a pivotal moment: embracing complexity—rather than simplifying it—may ultimately be the only way to achieve robust, integrated, and clinically meaningful osteochondral regeneration. Though variability in immune responses and long-term integration remain challenges, the ability to tailor hydrogel properties to patient-specific needs offers exciting opportunities for personalized regenerative therapies. Ultimately, embracing complexity is not a barrier but a necessary evolution: it empowers us to move beyond simplistic hydrogels toward truly adaptive, multifunctional platforms capable of guiding coordinated cartilage and bone healing. This complexity-driven approach, supported by ongoing technological and scientific advances, holds the greatest promise to overcome longstanding limitations and achieve durable, clinically meaningful osteochondral repair.

## Figures and Tables

**Table 1:** Summary of the advantages and disadvantages of the several types of hydrogels.

Category	Advantages	Disadvantages
Natural Hydrogels	Intrinsic biocompatibility and bioactivity. Innate biochemical motifs for cell interaction. Possibility of functionalization. Promote ECM synthesis.	Biological variability causes inconsistent batches and unpredictable degradation. Insufficient mechanical performance. Frequently combined in multilayered/hybrid constructs to improve mechanical strength.
Synthetic Hydrogels	Precise modulation of hydrogel mechanics, crosslinking density, and degradation kinetics. Promote ECM synthesis. High producibility and scalability. Possibility of functionalization.	The absence of inherent bioactivity requires the integration of bioactive additives. Degradation into acidic byproducts. May need reinforcement for long-term applications.
Hybrid and Composite Hydrogels	Complementary advantages of natural and synthetic hydrogels. Support cell adhesion, proliferation, and differentiation. Can be reinforced with inorganic fillers. Promote ECM synthesis. Possibility of functionalization.	Risk of phase separation. Possibility of filler dispersion. Difficult to ensure consistent crosslinking at layer interfaces. Increased fabrication complexity impacts on reproducibility and mechanical reliability.

## Data Availability

This review does not involve the generation or analysis of new data. Therefore, no data are available.
